# Prognostic Value of an Immunohistochemical Signature in Patients With Head and Neck Mucosal Melanoma

**DOI:** 10.3389/fimmu.2021.708293

**Published:** 2021-07-29

**Authors:** Qing-Qing Xu, Qing-Jie Li, Cheng-Long Huang, Mu-Yan Cai, Mei-Fang Zhang, Shao-Han Yin, Li-Xia Lu, Lei Chen

**Affiliations:** ^1^Department of Radiation Oncology, Sun Yat-Sen University Cancer Center, State Key Laboratory of Oncology in South China, Collaborative Innovation Center for Cancer Medicine, Guangdong Key Laboratory of Nasopharyngeal Carcinoma Diagnosis and Therapy, Guangzhou, China; ^2^Department of Pathology, Sun Yat-Sen University Cancer Center, Guangzhou, China; ^3^Imaging Diagnosis and Interventional Center, Sun Yat-Sen University Cancer Center, State Key Laboratory of Oncology in Southern China, Collaborative Innovation Center for Cancer Medicine, Guangzhou, China

**Keywords:** head and neck mucosal melanoma, immunohistochemistry, nomogram, prognosis, signature

## Abstract

**Purpose:**

We aimed to develop a prognostic immunohistochemistry (IHC) signature for patients with head and neck mucosal melanoma (MMHN).

**Methods:**

In total, 190 patients with nonmetastatic MMHN with complete clinical and pathological data before treatment were included in our retrospective study.

**Results:**

We extracted five IHC markers associated with overall survival (OS) and then constructed a signature in the training set (n=116) with the least absolute shrinkage and selection operator (LASSO) regression model. The validation set (n=74) was further built to confirm the prognostic significance of this classifier. We then divided patients into high- and low-risk groups according to the IHC score. In the training set, the 5-year OS rate was 22.0% (95% confidence interval [CI]: 11.2%- 43.2%) for the high-risk group and 54.1% (95% CI: 41.8%-69.9%) for the low-risk group (P<0.001), and in the validation set, the 5-year OS rate was 38.1% (95% CI: 17.9%-81.1%) for the high-risk group and 43.1% (95% CI: 30.0%-61.9%) for the low-risk group (P=0.26). Multivariable analysis revealed that IHC score, T stage, and primary tumor site were independent variables for predicting OS (all P<0.05). We developed a nomogram incorporating clinicopathological risk factors (primary site and T stage) and the IHC score to predict 3-, 5-, and 10-year OS.

**Conclusions:**

A nomogram was generated and confirmed to be of clinical value. Our IHC classifier integrating five IHC markers could help clinicians make decisions and determine optimal treatments for patients with MMHN.

## Introduction

Mucosal melanoma (MM) is rare in Caucasians, accounting for approximately 1.3% of all melanomas (1, 2), while it is the second most common subtype in China, accounting for 20%-25% of all melanomas (3, 4). The head and neck region has the highest incidence of MM, accounting for 55% of all cases (1). In China, MM of the head and neck (MMHN) represents approximately 1.7% of all head and neck malignancies, of which 94.56% occur in the mucosa. In China, MMHN mainly occurs in the nasal sinus and oral cavity. SEER database statistics (5) show that 72% of malignant melanomas occur in the nasal sinus, 19% occur in the oral cavity, and 9% occur in other areas, including the nasopharynx, oropharynx and larynx. MMHN is a malignant tumor with a poorer prognosis than other subtypes (3, 4, 6). According to the National Comprehensive Cancer Network (NCCN) guidelines (2019), surgical resection is preferentially recommended for localized MMHN (7). Immunotherapy and targeted therapy have recently changed the treatment landscape for MMHN (8, 9). However, these systemic therapies for MMHN have been evaluated only in small studies (10–13) or retrospective case studies (14–16). The gold standard for the diagnosis of MMHN is histopathological examination. Melanoma cells have a variety of phenotypes, including spindle, plasmacytoid, epithelial, etc. (17, 18). Tumor cells are arranged in lamellar, nest, and globoid structures at the microscopic level, and tumors with mixed cell phenotypes are more malignant than those with a similar phenotype throughout (17). Cells with cytoplasm rich in pigment granules make the disease easy to diagnose. However, approximately 13% ~ 25% of tumors lack melanin particles and need to be distinguished from other cancers, lymphomas, and sarcomas. Immunohistochemistry (IHC), an inexpensive and easy-to-use approach, is the most widely applied pathological technique in determining the expression of tumor-associated proteins. IHC analysis is often applied to distinguish different classifications of MMHN. Common immunohistochemical indicators include S⁃100, HMB⁃45 and Melan⁃A. It has been reported that many IHC-based markers can predict the prognosis of MMHN, but none have made it into clinical practice (19, 20). In addition, prognostic models incorporating multiple biomarkers help clinicians make treatment decisions and develop optimal treatment combinations to reduce disease mortality.

The objective of this study was to develop and validate an IHC-based classifier with a least absolute shrinkage and selection operator (LASSO) Cox regression model and to develop a prognostic nomogram for MMHN patients based on IHC biomarkers and clinicopathological factors.

## Materials and Methods

**Figure 1:**
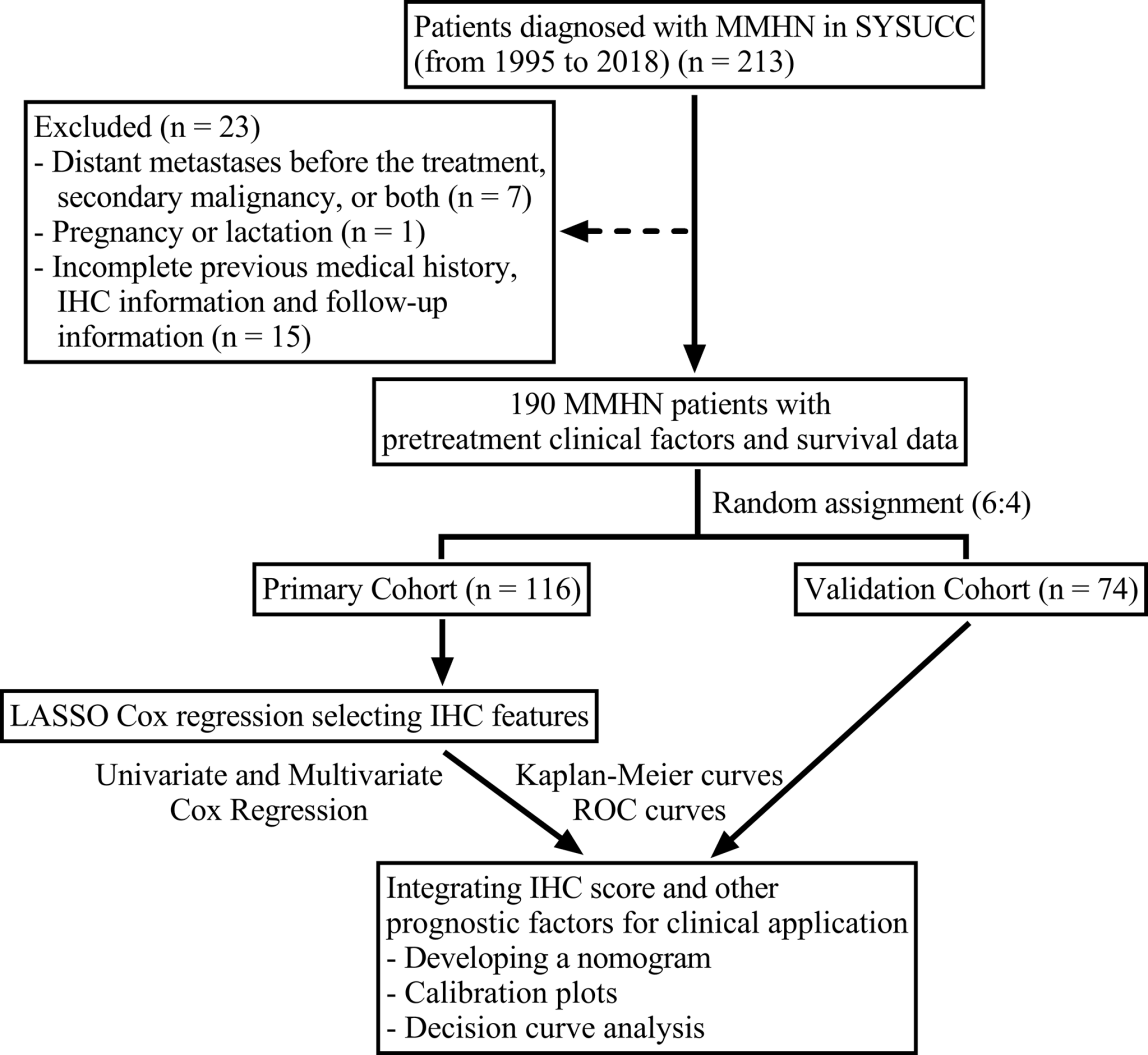
Flow chart of the study design. MMHN, mucosal melanoma of the head and neck; SYSUCC, Sun Yat-Sen University Cancer Center; IHC, immunohistochemistry; ROC, receiver operating characteristic.

A total of 190 previously untreated, nonmetastatic MMHN patients diagnosed at Sun Yat-Sen University Cancer Center between 1995 and 2018 were retrospectively included in the study. Patients were randomly assigned to the discovery cohort (116 cases) or validation cohort (74 cases) at a ratio of 6:4. The tumor-node-metastasis (TNM) stage of the patients was reassessed according to the American Type Tissue Culture Collection (AJCC) 8th edition guidelines (21). The study workflow of patient eligibility was displayed in **Figure 1**. The exclusion criteria were as follows: (1) evidence of distant metastasis before treatment, secondary malignancy, or both; (2) pregnancy or lactation; and (3) incomplete previous medical history, IHC information and follow-up information. The ethics committee of Sun Yat-Sen University Cancer Center approved our study protocol.

### Development and Validation of an Immunohistochemical Predictor

The LASSO Cox regression method was used to shrink the regression coefficients of the features and select the best combination of survival outcome predictors. Prognostic characteristics with nonzero coefficients were enrolled in the model. Each prognostic score was calculated according to a combination of these features. We developed a multimarker classifier to predict overall survival (OS) in patients with MMHN. The LASSO Cox regression model was applied by the “glmnet” package. We applied the survival R package to calculate the risk score, regarded as IHC score. (https://CRAN.R-project.org/package=survivalAnalysis). MMHN patients were divided into high- and low-risk groups based on the median value of the IHC score.

### Statistical Analysis

The chi-square test for categorical variables was used to assess differences between two groups. The survival time of patients separated into different risk groups according to the IHC signature score was estimated by Kaplan-Meier survival analysis with the log-rank test. We applied the ‘pROC’ package to perform receiver operating characteristic (ROC) analysis to investigate the prognostic properties of IHC features. The independent prognostic variables were analyzed by univariable and multivariable Cox regression analyses.

A nomogram was further constructed to predict the OS probability by accounting for the Cox regression coefficients. Calibration plots were drawn on the basis of the regression analysis results. Decision curve analysis (DCA) was performed to estimate the clinical utility. All statistical analyses were carried out with R software (version 4.0.3).

## Results

### Patient Characteristics

For the whole cohort, the median age was 56 (range, 19–87) years old, with a male to female ratio of 1.79:1. The primary tumor sites were as follows: The nasal cavity in 101 (53.2%) patients, the paranasal sinus in 10 (5.3%) patients, the oral cavity in 47 (24.7%) patients, and other sites such as the nasopharynx, oropharynx, eyelids, and larynx in 32 (16.8%) patients. The median follow-up time was 31 months (range, 2–206 months), during which there were 102 deaths. The baseline characteristics between the primary cohort and validation cohort are listed in **Table 1**.

**Table 1:** Baseline characteristics between the primary cohort and validation cohort.

	Training set		Validation set	
	Low‐risk patients (n = 75)	High‐risk patients (n = 41)	Low‐risk patients (n = 62)	High‐risk patients (n = 12)
Gender				
Male	51 (68.00%)	26 (63.41%)	36 (58.06%)	9 (75.00%)
Female	24 (32.00%)	15 (36.59%)	26 (41.94%)	3 (25.00%)
Age (year-old)				
<= 60	45 (60.00%)	27 (65.85%)	39 (62.9%)	7 (58.33%)
> 60	30 (40.00%)	14 (34.15%)	23 (37.1%)	5 (41.67%)
Tumor site				
Others	8 (10.67%)	8 (19.51%)	16 (25.81%)	0
Nasal cavity	45 (60.00%)	18 (43.90%)	32 (51.61%)	6 (50.00%)
Paranasal sinus	3 (4.00%)	4 (9.76%)	3 (4.84%)	0
Oral cavity	19 (25.33%)	11 (26.83%)	11 (17.74%)	6 (50.00%)
T stage				
T3	47 (62.67%)	15 (36.59%)	28 (45.16%)	6 (50.00%)
T4	28 (37.33%)	26 (63.41%)	34 (54.84%)	6 (50.00%)
N stage				
N0	58 (77.33%)	27 (65.85%)	44 (70.97%)	6 (50.00%)
N1	17 (22.67%)	14 (34.15%)	18 (29.03%)	6 (50.00%)

### Immunohistochemical Signature Development

**Figure 2:**
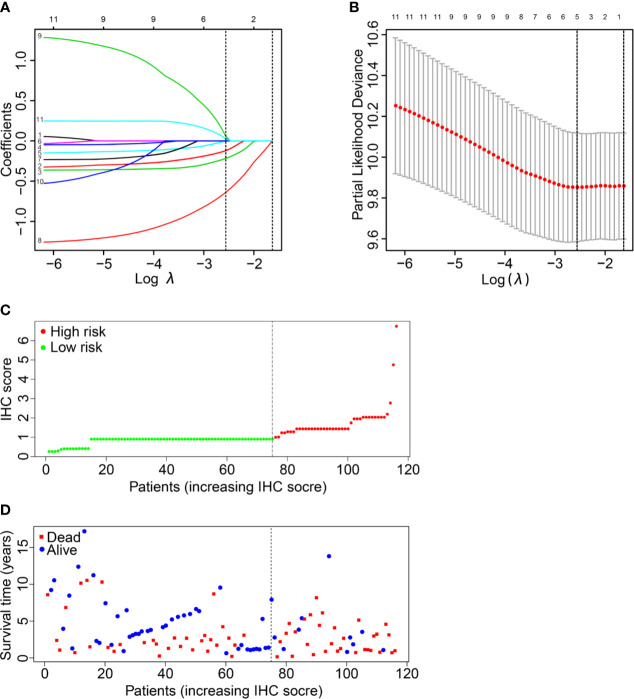
IHC feature selection using the LASSO cox regression model. **(A)** A LASSO coefficient profile plot of the IHC features was produced against the logλ sequence. **(B)** Ten-fold cross-validation for IHC features selection in the LASSO Cox model. The dotted vertical lines represented the λ values with minimal deviance (left) and with the largest λ value within one standard deviation of the minimal deviance (right). We then selected the coefficients of the model with the minimal deviance, which included 5 IHC features. **(C, D)** The IHC score classified MMHN patients into low-risk and high-risk groups.

**Figure 3:**
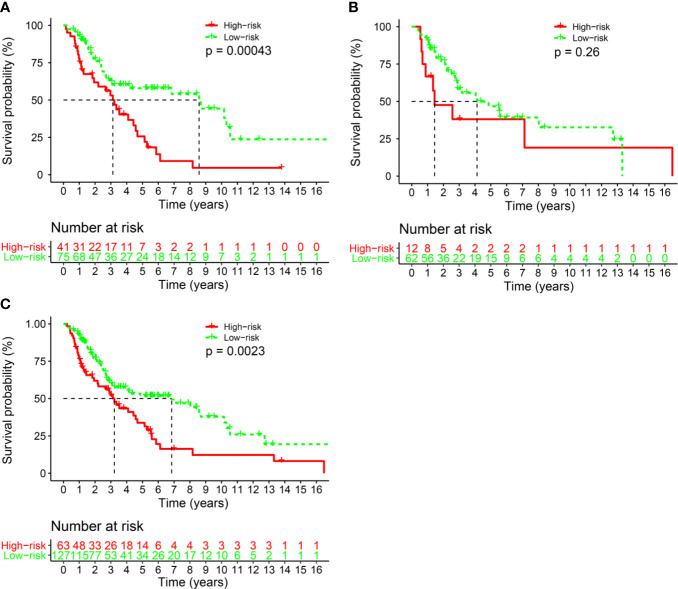
The comparison of overall survival between the high- and low-risk groups stratified by IHC score. **(A)** The Discovery cohort, **(B)** the validation cohort, and **(C)** the total cohort.

Eleven IHC features (S100, HMB45, Melan-AC, Ki67, Vim, SOX-10, CD56, CK, Syn, CgA1, NSE) were reduced to five prognostic markers (HMB45, Melan-AC, CK, Syn, NSE) in the training set *via* the LASSO Cox regression model (**Figures 2A, B** **)**. Based on the median IHC score calculated with the R survival package, we divided patients of the discovery cohort into low-risk and high-risk groups. The distribution plot of IHC score showed that the risk of mortality increased with increasing IHC score **(** **Figures 2C, D** **)**. The high-risk group (41 patients, 35.3%) included patients with an IHC score of 0.9 or higher, while the low-risk group (75 patients, 64.7%) included patients with an IHC score below 0.9. Patients in the low-risk group had a better 5-year OS rate [54.1% (95% confidence interval (CI): 41.8%-69.9%)] than those in the high-risk group [22.0% (95% CI: 11.2%- 43.2%); P<0.001; **Figure 3A**].

### Validation of the Signature

We also conducted the same analysis in the validation set (n=74). Patients were divided into a low-risk group (62 patients, 83.8%) and a high-risk group (12 patients, 16.2%) according to the IHC score. The 5-year OS rate was 43.1% (95% CI: 30.0%-61.9%) in the low-risk group and 38.1% (95% CI: 0.18–0.8117.9%-81.1%; P= 0.26) in the high-risk group **(** **Figure 3B** **)**. There was a statistically significant difference between the two risk groups in the whole cohort (P<0.001, **Figure 3C**).

### Prediction Accuracy of the IHC Classifier

**Figure 4:**
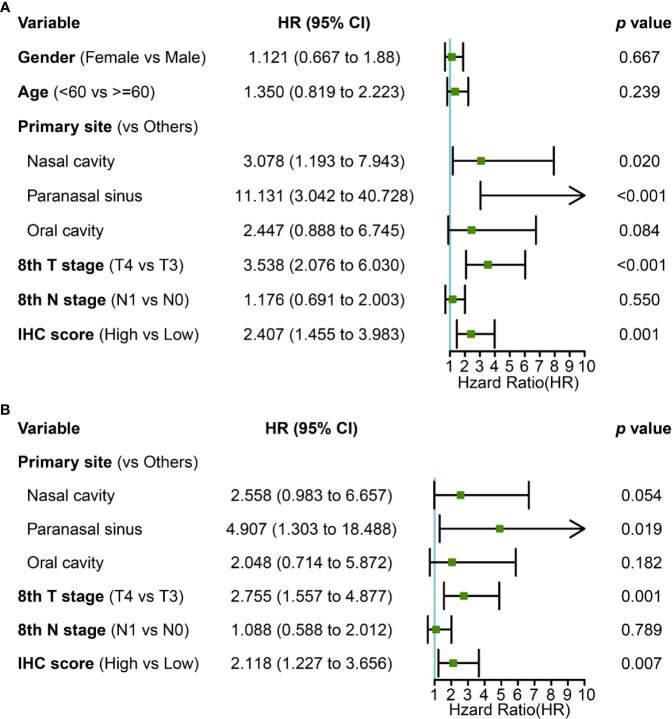
**(A)** univariate and **(B)** multivariate Cox regression with clinical information and IHC score for OS.

**Figure 5:**
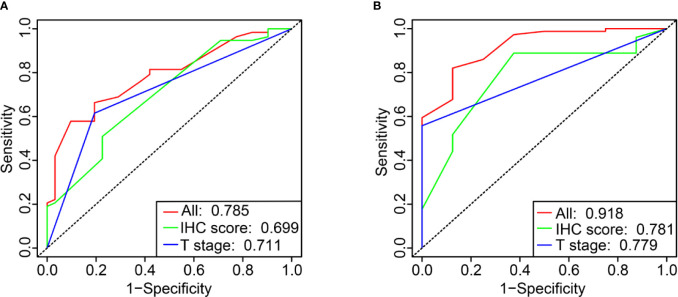
Time-dependent ROC curve comparing the prognostic value of IHC score in 5 and 10 years with that of T stage **(A, B)**.

Primary tumor site, T stage, and IHC score were significant prognostic variables in the univariable analysis (**Figure 4A**). Primary tumor site, T stage, and IHC score remained independent predictors in multivariate analysis (**Figure 4B**). In addition, receiver operating characteristic (ROC) curve analysis of 5- and 10-year OS showed that the area under the receiver operating characteristic curve (AUROC) values for the IHC score was 0.699 and 0.781, respectively, which was similar to that of T stage (P= 0.866 and 0.979). Furthermore, the combination of IHC score and T stage was superior in predicting 5- and 10-year OS than T stage alone (5-year OS, P = 0.013; 10-year OS, P = 0.005). Thus, IHC score could contribute to predicting the 5- and 10-year OS (**Figures 5A, B** **)**.

### Nomogram Building and Clinical Utility Assessment

**Figure 6:**
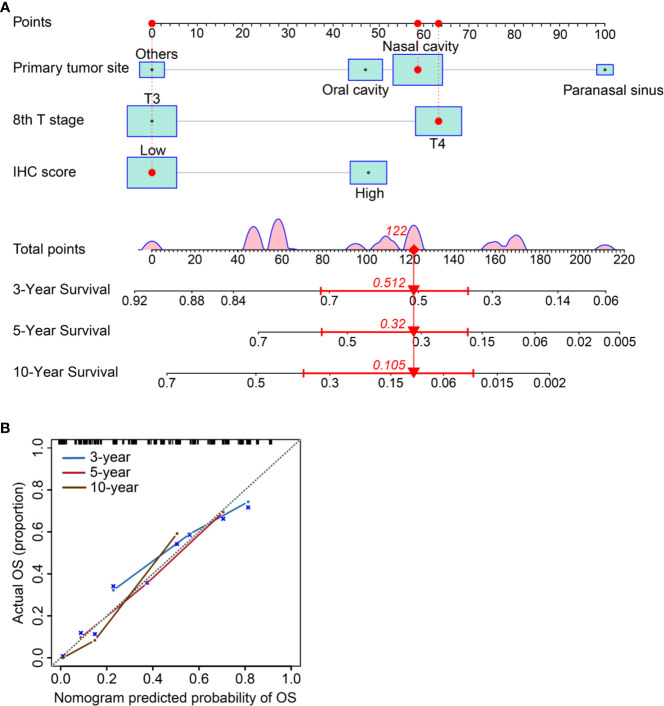
Establishing and validating a nomogram. **(A)** A constructed nomogram for prognostic prediction of a patient with MMHN. The patient had a local tumor in nasal cavity, T4 stage, and low IHC score. Density plot of total points shows their distribution. For category variables, their distributions are reflected by the size of the box (to view boxes of IHC score, the smaller one represents high score and the bigger one represents low). The importance of each variable was ranked according to the standard deviation along nomogram scales. To make use of the nomogram, the black dots of a single patient are located on each of the variable axis. Red dots and lines are drawn up to determine the points received by each variable; the sum (122) of these points is located on the Total Points axis, and a line is drawn downward to the survival axes to determine the probability of 3-year (51.2%), 5- year (32.0%) and 10-year (10.5%) overall survival for MMHN. **(B)** The 3-, 5-, and 10-year OS of calibration curves were used to assess the predictive ability of the nomogram. Dashed lines along the 45-degree line represented that the predicted probabilities are equal to the actual probabilities.

**Figure 7:**
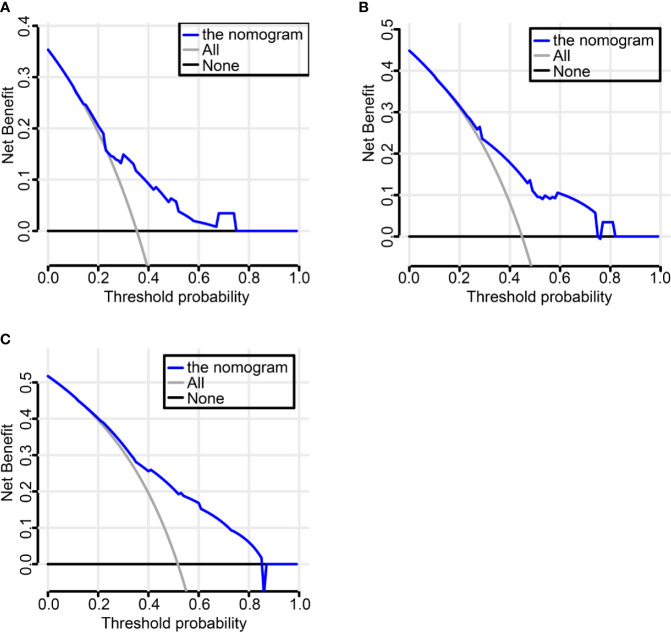
Decision Curve Analysis (DCA) to evaluate clinical utility of the nomogram. The y-axis measures the net benefit. The dashed line represents the nomogram. Using the IHC-based nomogram provides a better net benefit than all or no treatment **(A–C)**.

We established a nomogram incorporating IHC score, T stage, and primary tumor site and its prognostic efficacy (**Figure 6A**). The nomogram displays an example of a given patient to predict survival probability. The total score was depended on each scores calculated by the nomogram; the total risk point for most patients in this study was from 0 and 220. Calibration curves showed high consistency between the Kaplan-Meier estimates and those from our nomogram in both data sets (**Figure 6B**). Finally, the clinical value of the nomogram was evaluated by DCA. The nomogram has promising clinical value because the range of the 3-, 5-, and 10-year threshold probabilities for OS indicate that the nomogram provides a better net benefit than all or no treatment (**Figures 7A–C**).

## Discussion

Current treatment modalities do not provide clinically useful molecular prognostic biomarkers. We developed a nomogram for predicting 3-, 5-, and 10-year OS in patients with MMHN by combining useful immunohistochemical biomarkers and clinical features; this model can help clinicians make treatment decisions. A previous study reported the function of the biomarkers found in our study. The expression of Melan-AC is highly specific in differentiating lymph nodes from metastatic melanoma (22), whereas the prognostic function of Melan-AC remains unknown. Syn is abnormally regulated in many cancers and plays an important role in the proliferation and progression of tumor cells (23). Syn is a typical pathological amyloid protein observed in melanoma, whose expression is negatively correlated with the melanin content (24). However, the prognostic function of syn remains controversial. It has been reported that increased cytokeratin (CK) expression is connected with highly malignant melanoma (25). Our results showed that CK is expressed in many advanced unresectable metastatic melanomas. However, the prognostic role of CK in patients with MMHN remains controversial. HMB-45 plays an important role in diagnosing amelanotic melanoma (26). It could also be a good marker for diagnosing primary oral and nasal MM (27). The expression of HMB-45 appears to be highly specific for diagnosis in MM (28), but the prognostic function of HMB-45 has not been reported. NSE may be a potential target for lymphoma therapy and a prognostic marker for lymphoma (29). Patients with high expression of NSE for lymphoma had worse clinical outcomes than those with low expression of NSE. However, the tumor-promoting mechanism of NSE and the prognostic role of MMHN remain unclear. The expression of NSE could be helpful for disease assessment and for the early identification of distant metastases in patients with melanoma (30).

To our knowledge, this is the first nomogram constructed to predict the OS probability of patients with MMHN. In this study, we successfully integrated multiple immunohistochemical markers into a single signature *via* a LASSO Cox regression model, and this signature had significantly higher prognostic accuracy than a single immunohistochemical marker alone. With this model, the 3-, 5-, and 10-year OS of individual patients with MMHN can be predicted accurately. For example, a patient with T3 stage disease (0 points), paranasal sinus MM (100 points), and a low IC score (0 points) would have a total score of 100 points, yielding an estimated 3-year OS rate of 62%.

The main limitation of our study is that the nomogram is based on retrospective data from a single cancer center. Further multicenter, prospective clinical trials are needed to verify our results.

## Conclusions

We developed and validated a nomogram incorporating clinicopathological characteristics and IHC features that can precisely predict the prognosis of patients with MMHN and help clinicians develop an optimal treatment strategy.

## Data Availability Statement

The raw data supporting the conclusions of this article will be made available by the authors, without undue reservation.

## Ethics Statement

The studies involving human participants were reviewed and approved by The ethics committee of Sun Yat-Sen University Cancer Center. The patients/participants provided their written informed consent to participate in this study.

## Author Contributions

Study conceptualization (LC and L-XL). Data curation and investigation (Q-QX, LC, and L-XL). Funding acquisition (LC and L-XL). Data analysis, interpretation and visualization (Q-QX, Q-JL, and C-LH). Quality control of data and algorithms (M-YC, M-FZ, and S-HY). Manuscript writing (Q-QX, Q-JL, and C-LH). Manuscript reviewing and approving (Q-QX, Q-JL, LC, and L-XL). All authors contributed to the article and approved the submitted version.

## Funding

This study was funded by the Planned Science and Technology Project of Guangdong Province (No 2016A020215085, 201707010087) and the 308 Clinical Research Funding of Sun Yat-Sen University Cancer Center (No 308-2015-011). These funding sources had no role in the study design, data collection, data analysis, interpretation, and writing of the report.

## Conflict of Interest

The authors declare that the research was conducted in the absence of any commercial or financial relationships that could be construed as a potential conflict of interest.

## Publisher’s Note

All claims expressed in this article are solely those of the authors and do not necessarily represent those of their affiliated organizations, or those of the publisher, the editors and the reviewers. Any product that may be evaluated in this article, or claim that may be made by its manufacturer, is not guaranteed or endorsed by the publisher.
